# Evaluating Relationships between Wild Skeena River Sockeye Salmon Productivity and the Abundance of Spawning Channel Enhanced Sockeye Smolts

**DOI:** 10.1371/journal.pone.0095718

**Published:** 2014-04-23

**Authors:** Michael H. H. Price, Brendan M. Connors

**Affiliations:** 1 SkeenaWild Conservation Trust, Terrace, British Columbia, Canada; 2 ESSA Technologies Ltd., Vancouver, British Columbia, Canada; 3 School of Resource and Environmental Management, Simon Fraser University, Burnaby, British Columbia, Canada; North Carolina State University, United States of America

## Abstract

The enhancement of salmon populations has long been used to increase the abundance of salmon returning to spawn and/or to be captured in fisheries. However, in some instances enhancement can have adverse impacts on adjacent non-enhanced populations. In Canada's Skeena watershed, smolt-to-adult survival of Babine Lake sockeye from 1962–2002 was inversely related to the abundance of sockeye smolts leaving Babine Lake. This relationship has led to the concern that Babine Lake smolt production, which is primarily enhanced by spawning channels, may depress wild Skeena (Babine and non-Babine) sockeye populations as a result of increased competition between wild and enhanced sockeye smolts as they leave their natal lakes and co-migrate to sea. To test this hypothesis we used data on Skeena sockeye populations and oceanographic conditions to statistically examine the relationship between Skeena sockeye productivity (adult salmon produced per spawner) and an index of Babine Lake enhanced smolt abundance while accounting for the potential influence of early marine conditions. While we had relatively high power to detect large effects, we did not find support for the hypothesis that the productivity of wild Skeena sockeye is inversely related to the abundance of enhanced sockeye smolts leaving Babine Lake in a given year. Importantly, life-time productivity of Skeena sockeye is only partially explained by marine survival, and likely is an unreliable measure of the influence of smolt abundance. Limitations to our analyses, which include: (1) the reliance upon adult salmon produced per spawner (rather than per smolt) as an index of marine survival, and (2) incomplete age structure for most of the populations considered, highlight uncertainties that should be addressed if understanding relationships between wild and enhanced sockeye is a priority in the Skeena.

## Introduction

Artificial propagation (enhancement) has become an important tool for maintaining the harvest of exploited fish species [1], and such programs are increasingly being initiated to restore threatened and endangered wild populations [2], [3]. Whether or not to use enhancement programs, such as fish hatcheries, is a pressing issue for the conservation of Pacific salmon (*Oncorhynchus* spp.) because it remains unclear whether these programs in fact aid the recovery of depressed wild populations [4]–[6]. There is mounting evidence that suggests fish hatcheries can have unintended genetic and ecological consequences for wild salmon, leading to reduced productivity and abundance [7]–[10]. One such consequence is a density-dependent decrease in survival during early marine residency due either to an increase in competition for food resources [11] or the functional response of predators [12]. Furthermore, large-scale hatchery programs have effectively replaced wild salmon with hatchery salmon in many areas of the Pacific [13]. Much less understood, however, is the potential negative influence of salmon enhancement from human-created (artificial) spawning channels on wild salmon.

**Figure 1:**
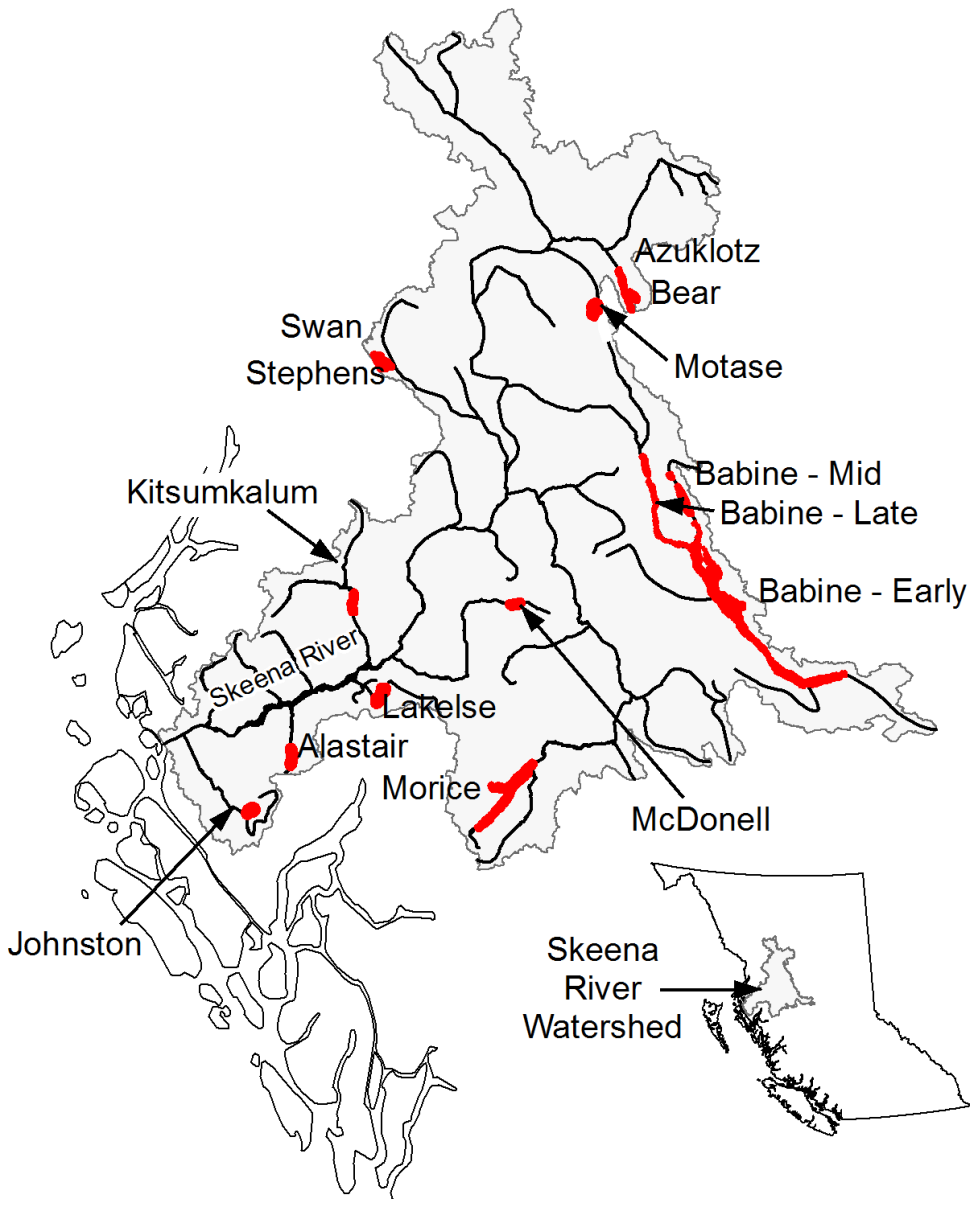
Skeena River catchment lake-sockeye Conservation Units included in our analysis. Legend: Red-areas are sockeye nursery lakes. Map is adapted from [14].

Canada's largest (by area) sockeye salmon (*O*. *nerka*) producing lake system, Babine-Nilkitkwa in the Skeena River watershed (Figure 1), hosts three artificial spawning channels. Sockeye spawn naturally in the channels, but progeny share the freshwater lake system and at least some portion of coastal marine rearing habitats with numerous wild sockeye populations from the Skeena watershed and beyond [14], [15]. Built in the 1960s, the spawning channels have been successful at increasing the number of sockeye that return to Babine Lake annually [14], [16]. Fry recruitment to the Main Arm of Babine Lake has increased three-fold since completion of the spawning channels [17], from an average 61 million annually during 1950–1970 to an average 192 million annually since then; fry recruitment from the spawning channels now accounts for approximately 91% of all Babine Lake fry compared to 67% prior to 1970 [15]. Despite the success in increasing fry recruitment, it has been suggested that sockeye enhancement in Babine Lake may adversely impact wild sockeye from Babine Lake and other Skeena nursery lakes in three ways: i) overfishing in non-selective marine fisheries that target productive enhanced sockeye [14], [18], ii) pathogen transfer [19], and iii) increased competition between wild and enhanced sockeye smolts as they leave their natal lakes and co-migrate to sea [20], [21].

**Figure 2:**
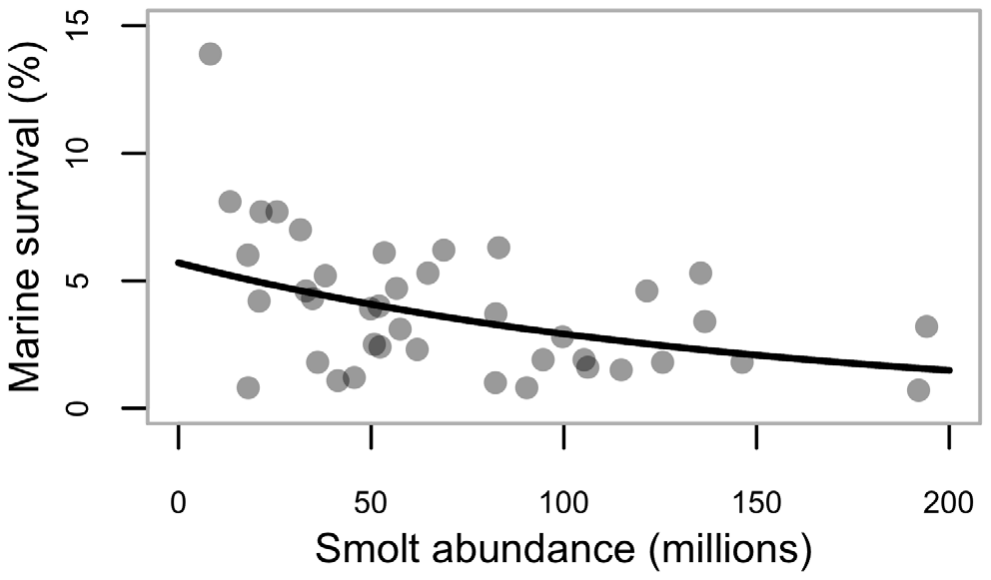
Relationship between sockeye smolt-to-adult survival and smolt abundance. Legend: Aggregate Babine (i.e., wild and enhanced) sockeye smolt-to-adult survival as a function of smolt abundance (the total number of out-migrating sockeye smolts in a given year between 1962–2002). Line is the best-fit relationship based on a nonlinear least-squares model fit (y = 7.4exp[−0.01*x], *p* = 0.0013).

There are several reasons to assess whether enhanced (which herein refers to enhancement from spawning channels) sockeye from Babine Lake negatively influence wild Skeena sockeye. First, a report by the Skeena Independent Science Review Panel recommended a formal assessment of the impact of enhanced stocks on wild stocks within the same Canadian sockeye salmon system [21]. Second, the recent certification of the Skeena commercial sockeye fishery as *sustainable* by the Marine Stewardship Council is conditional (in part) “…until a peer-reviewed assessment of the impact of production from spawning channels on wild sockeye stocks has been completed…” [22]. An inverse relationship between smolt-to-adult survival for combined Babine sockeye populations and the abundance of smolts leaving Babine Lake in a given year was first described by Peterman [20]. This relationship continued to hold through 2002, when efforts to estimate out-migrating smolt abundance in Babine Lake ceased [15], [17]; our Figure 2. The survival of wild non-Babine Skeena sockeye that migrate to sea with channel-enhanced smolts may also be reduced in years when the number of enhanced smolts is high [21]. However, to date there has not been an empirical examination of this relationship because data on wild Skeena sockeye populations, including smolt-to-adult survival, have not been available.

Recently reconstructed stock-recruitment time series for wild Skeena sockeye Conservation Units (CUs; Canada's analogue to Evolutionarily Significant Units — ESUs — in the U.S.A.) now make it possible to begin to explore relationships between smolt abundance and wild Skeena sockeye productivity [23], [24]. We used data on Skeena sockeye salmon populations and oceanographic conditions to ask the question: is wild Skeena sockeye productivity (adult recruits produced per spawner) negatively related to the abundance of enhanced sockeye smolts leaving Babine Lake, and are the effects of competition between enhanced and wild sockeye greatest in years when conditions in early marine life are least favorable? The analyses we describe provide a quantitative foundation upon which these relationships can begin to be examined and understood, and we offer suggestions to address current data gaps.

## Methods

### Sockeye data

We considered estimates of sockeye spawner abundance and exploitation rates for Skeena sockeye CUs [23], [24], which were downloaded from the Pacific Salmon Foundation's Skeena Salmon Program website (www.skeenasalmonprogram.ca). This dataset includes time-series of sockeye spawner abundance from 16 lake-sockeye CUs and three wild Babine Lake run-timing groups. Time-series for each CU and run-timing group were of differing length. Although there also are several Skeena river-type sockeye CUs, none had sufficient data to be included in our analysis. Adult recruits from each brood year in each CU and group were estimated based on the assumed age structure for each CU in each year (see below) and estimates of total exploitation rates by return year [24]. To generate stock-recruit data for wild Babine Lake sockeye, the Babine Lake stock-recruit time-series [23] was separated into wild (run timings: early, mid, and late) and enhanced components based on estimates of each component (see below) [15].

Age composition data were available for every year of the Babine Lake time-series (which we assume are identical across run-timings), but not for any other Skeena lake-sockeye CU. We used average age compositions across years for CUs where there was not age composition data for each year but at least some age composition data. We used the average age composition across years from neighboring CUs thought to have similar age composition [23] for those CUs without any age composition data (Table 1). Only brood years with corresponding recruitment that comprised 95% or more of the known or assumed age composition were used in this analysis. The resulting dataset consisted of 11 wild Skeena lake-sockeye CUs, plus 3 wild Babine Lake run-timing groups (Table 1).

**Table 1:** Wild Skeena lake sockeye Conservation Units (CU) and Babine Lake sockeye run-timing groups used in the analyses (CU/group).

CU/group	Average spawners (SD)	Average recruits (SD)	Stock-recruit	First/last year	Age-samples	Age-years
Alastair	15,569 (9,026)	25,808 (14,609)	38	1960/2000	151	2
Azuklotz	3,449 (2,475)	6,620 (4,523)	16	1960/2000	0	0
Babine (early)*	55,362 (31,398)	124,228 (113,056)	40	1960/2000	17,489	21
Babine (late)*	255,186 (145,265)	681,660 (504,604)	40	1960/2000	17,489	21
Babine (mid)*	18,378 (13,248)	45,264 (37,672)	40	1960/2000	17,489	21
Bear	1,293 (998)	3,870 (3,849)	12	1960/1993	46	1
Johnston	4,858 (5,351)	7,148 (4,832)	12	1965/1997	0	0
Kitsumkalum	4,695 (4,565)	15,266 (14,932)	36	1960/2000	0	0
Lakelse	17,677 (17,049)	23,747 (19,106)	33	1960/1992	194	1
McDonell	3,068 (2,497)	5,165 (2,356)	21	1960/1982	0	0
Morice	15,243 (23,542)	50,193 (70,700)	34	1960/1998	98	1
Motase	531 (455)	1,041 (570)	7	1992/2000	0	0
Stephens	6,580 (3,356)	13,426 (4,587)	27	1960/1999	0	0
Swan	7,683 (5,527)	16,543 (11,309)	17	1960/1999	100	1

*The number of stock-recruitment pairs, age-samples, and age-years are shared among the 3 wild Babine groups.

Legend: “Stock-recruit” is the total number of stock-recruitment pairs available for each CU/group between 1960 and 2000 brood years, “First/last year” is the first and last brood year of the stock-recruit time series for each CU/group used in the analyses, “Age-samples” is the total number of adult age samples for the CU/group, and “Age-years” is the number of years where age data was available.

**Figure 3:**
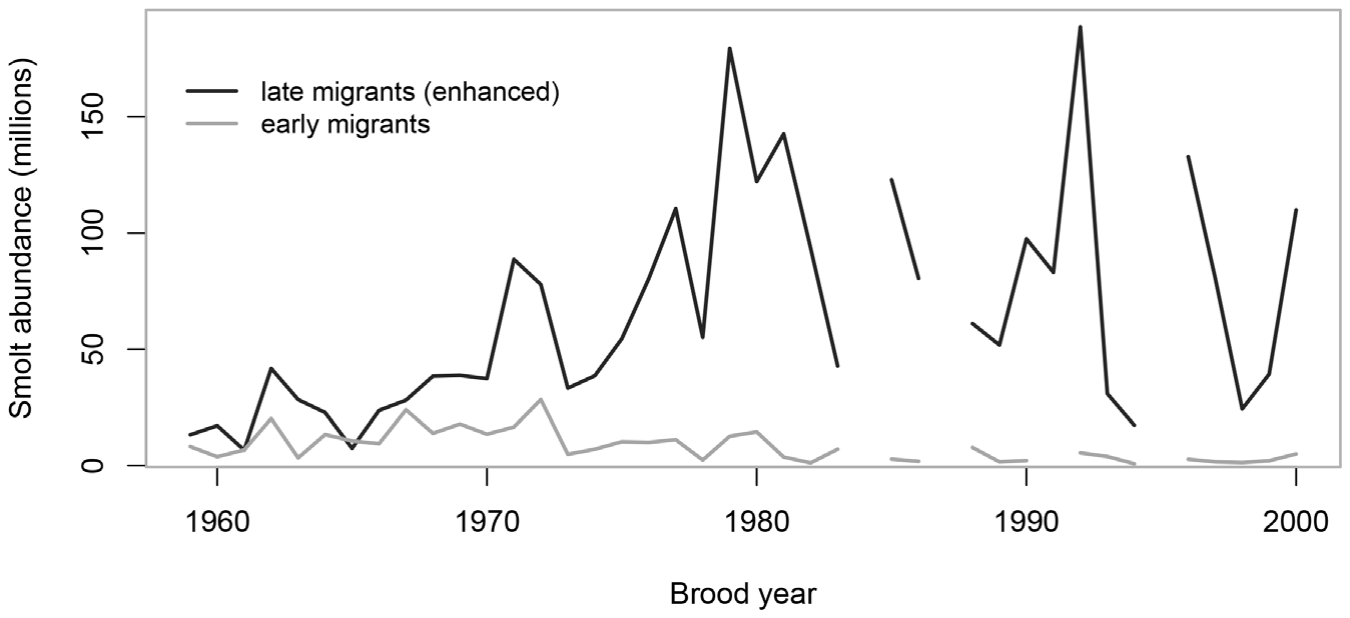
Babine Lake sockeye smolt abundance over time. Legend: Abundance of late-migrant (primarily channel-enhanced) and early-migrant (primarily wild) sockeye salmon smolts leaving Babine Lake as enumerated by smolt trap by sockeye brood year. Note: the smolt trap ceased to operate in 2002 (2000 brood year).

A mark-recapture program designed to estimate the total number of smolts leaving Babine Lake was operated almost continuously from 1959 to 2002 (Figure 3). The time-series of smolt abundance that resulted from these efforts is categorized into early and late migrants with late migrant smolts accounting for the majority of smolts leaving Babine Lake in a given year. Early migrant smolts are thought to primarily originate from late-timed adults spawning in the upper and lower Babine River, and late migrant smolts are thought to primarily originate from early- and mid-timed adults [17]. Early migrants typically accounted for less than 10% of total outmigrating smolt abundance in a given year since the mid-1970s. We used the abundance of late migrant smolts as an index of enhanced smolt abundance because they are predominately fish that are produced from channel-enhanced sockeye [15]. Smolt abundance in 1986 and 1997 were treated as missing data because estimates in those years are considered highly questionable [17].

### Oceanographic data

We calculated average sea surface temperature (SST, in °C) anomalies off the coast of the Skeena estuary from January to May in the year of sockeye marine entry to examine the influence of oceanographic conditions encountered by Skeena sockeye soon after entering the ocean in our analysis. Sea surface temperature is considered a proxy for the biological conditions experienced by salmon early in marine life. Sea surface temperature in the winter preceding marine entry is positively correlated with Skeena sockeye survival [25], [26], and is a stronger predictor of sockeye survival than SST during the spring and summer of the first year at sea [27], as well as upwelling indices, sea surface salinity, and larger-scale climate anomalies associated with the Pacific Decadal Oscillation [25], [26]. Sea surface temperature was compiled from NOAA reconstructed SST time-series for 2^O^ latitude-by-longitude cells (www.esrl.noaa.gov/psd) that encompassed the marine entry point of Skeena sockeye CUs and Babine run-timing groups.

### Hypotheses

We relied on an index of survival (adult recruits produced per spawner), which integrates over freshwater and saltwater life stages, to test the hypothesis that enhanced smolt production negatively impacts wild Skeena sockeye survival. While indices of survival separated by life stage (e.g., smolt-to-adult survival) would allow for a more powerful test, smolt abundance estimates for wild Skeena CUs have not been collected (with the exception of two CUs with less than 10 years of smolt data, and limited corresponding brood years). For Babine Lake (which includes channel-enhanced fish) where we do have estimates, smolt-to-adult survival and adult recruits produced per spawner are only moderately correlated (Pearson's r = 0.49, *p* = 0.001).

Our general hypothesis is: (i) wild Skeena sockeye productivity is negatively related to the number of enhanced sockeye smolts leaving Babine Lake, and (ii) the negative influence of high enhanced smolt abundance on wild Skeena sockeye productivity is greatest in years when conditions in early marine life are least favorable, and hence competition among smolts for resources is expected to be highest. We considered this general hypothesis at two spatial scales: 1) Skeena watershed, and 2) Babine Lake. Support for the hypothesis at the scale of the entire Skeena system would suggest that among population density-dependent interactions between enhanced and wild Skeena sockeye negatively influence all wild Skeena sockeye (for which there are data), while support at the scale of only Babine Lake would suggest that density-dependent competitive interactions only occur among sockeye that rear and emigrate from Babine Lake. The general hypothesis described above can be distilled into eight specific hypotheses (Table 2), which can be formulated as statistical models whose support can be quantified by confronting them with the available data.

**Table 2:** The eight specific hypotheses formulated as statistical models.

#	Hypotheses
1	Sockeye productivity across all Skeena CUs is inversely related to the abundance of
	enhanced Babine Lake smolts in the year wild sockeye migrate to sea.
2	Sockeye productivity from wild Babine Lake groups is inversely related to the abundance
	of enhanced Babine Lake smolts in the year wild sockeye migrate to sea (but productivity
	of CUs for other wild Skeena sockeye populations is not).
3	Sockeye productivity across all Skeena CUs is inversely related to the abundance of
	enhanced Babine Lake smolts in the year wild sockeye migrate to sea, and positively
	related to SST in the months preceding marine entry.
4	Sockeye productivity from wild Babine Lake groups is inversely related to the abundance
	of enhanced Babine Lake smolts in the year wild sockeye migrate to sea (but
	productivity of CUs for other wild Skeena sockeye populations is not), and positively
	related to SST (for all Skeena populations) in the months preceding marine entry.
5	Sockeye productivity across all Skeena CUs is inversely related to the abundance of
	enhanced Babine Lake smolts in the year wild sockeye migrate to sea, positively related
	to SST in the months preceding marine entry, and the relationship between smolt
	abundance and productivity is stronger in years when SST is low.
6	Sockeye productivity from wild Babine Lake groups is inversely related to the abundance
	of enhanced Babine Lake smolts in the year wild sockeye migrate to sea (productivity of
	CUs for other wild Skeena sockeye populations is not), positively related to SST in the
	preceding marine entry, and the relationship between smolt abundance and productivity
	is stronger in years when SST is low.
7	Sockeye productivity across all Skeena CUs is positively related to SST in the months
	preceding marine entry.
8	Sockeye productivity is not related to enhanced Babine Lake smolt abundance or SST.
	This null model is simply a model that includes within-CU density-dependence (i.e., a
	classic Ricker model).

### Statistical framework

To test the eight hypotheses, each hypothesis was formulated as a modified version of the Ricker stock-recruit relationship [28]. The full model (i.e., all hypotheses combined) is:
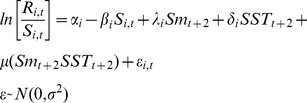
(1)where *R_i_*_,*t*_ is the total number of adult recruits to CU *i* produced by spawners (*S_i_*_,*t*_) in year *t*, *α* is the intrinsic rate of population growth (i.e., productivity at low spawner abundance) in CU *i*, *β* is density dependence in relation to the carrying capacity of CU *i*, *λ* and *δ* are the influence of Babine late-migrant smolt abundance and SST on sockeye productivity respectively, *μ* is the interaction term between SST and smolt abundance, and *ε_i_*_,*t*_ is residual error. Both smolt abundance and SST were lagged by 2 years (*t+2*; to account for eggs in gravel (year-1), and freshwater rearing (year-2)) to reflect conditions in the year of migration to sea for those CUs/groups where it was assumed sockeye migrate to sea in their second year of life (Babine groups, Lakelse, Johnston, and Stephens CUs). For all remaining CUs, enhanced Babine smolt abundance and SST were treated as the weighted average across the years that sockeye are thought to migrate sea (see Table 3 in [29]).

**Table 3:** Model selection statistics for the hypotheses considered ordered by small-sample Akaike Information Criteria differences from the top model (ΔAIC_c_).

#	Model	LogLik	ΔAICc	w*_i_*	Evidence ratio
8	Null	−451.49	0	0.59	-
7	SST	−450.14	1.16	0.33	1.79
1	Sm(f)	−451.48	4.66	0.06	9.83
3	Sm(f) + SST	−450.06	6.04	0.03	19.67
5	Sm(f) + SST + (Sm(f) x SST)	−449.43	10.94	0	inf
2	Sm(b)	−457.64	21.41	0	inf
4	Sm(b) + SST	−455.47	22.35	0	inf
6	Sm(b) + SST + (Sm(b) x SST)	−455.35	35.8	0	inf

Legend: Model terms are: enhanced Babine smolt abundance at the full Skeena (Sm(f)) and Babine Lake (Sm(b)) scale, sea surface temperature (SST), and an interaction between the two (x). “Null” is the null hypothesis, “LogL” is log likelihood, and “w*_i_*” is Akaike model weight. The evidence ratio [ratio of w_i_ values (for the best model divided by another model's w_i_)] is a measure of how much less likely a model is compared to the top model given the data and set of models considered. The number to the left of each model corresponds to the numbering of hypotheses in the main text, and “inf” means to infinite.

As opposed to examining the relationship between the abundance of enhanced Babine Lake smolts and wild Skeena River sockeye CU productivity on a CU-by-CU basis, all CUs were considered simultaneously. This approach looks for commonality in the response of each CU to smolt abundance and SST, which increases the chance of finding true relationships by allowing for common responses to be more easily isolated from random demographic noise and sampling errors [30], [31]. To do this, equation 1 was modified to:
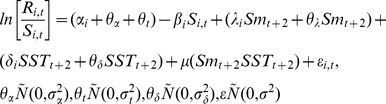
where *α*, *λ*, and *δ* are now shared responses (i.e., common to all the CUs) with additional additive stochastic CU-specific deviations, *θ*s, which are normally distributed with a mean of zero and variance *σ^2^* and covariance that is estimated [32]. In mixed-effects model terminology the *θ*s are random effects that capture CU-specific deviations from the shared response among CUs to smolt abundance and SST. The interaction term, *μ*, was estimated as common to all CUs but without CU-specific deviations because models with a random effect for the interaction failed to converge. Among-CU (*θ_α_*) and among-year (*θ_t_*) variability in *α* was also included in the model to account for variation in the intrinsic rate of population growth across CUs and across years.

The full model in equation 2, as well as reduced models representing the eight hypotheses (Table 2), were fitted to the data by maximum likelihood. Support for each of the hypotheses was quantified by small-sample Akaike Information Criteria (AIC_c_) [33]. To account for model (i.e., hypothesis) uncertainty, parameter estimates were re-estimated using restricted estimate maximum likelihood [34], and a weighted average of the parameter estimates based on model uncertainty (i.e., Akaike weights) was calculated according to the “natural average” method [35].

In addition to the statistical support for the models that represented each hypothesis, we also evaluated each hypothesis based on the direction and magnitude of parameter values that estimated the influence of each hypothesized factor on Skeena sockeye productivity (i.e., parameter estimates). All parameters were estimated from a dataset in standard deviation units (mean of zero, SD  = 1) to permit meaningful comparisons among parameters because the independent variables are on different numerical scales. All analyses were performed in R (version 2.15.1) using the lme4 (for the linear mixed-effects modelling) and MuMIn (for multi model inference) packages [36].

### Power analyses

To quantify the power that our model had to detect a specified effect size of Babine smolt abundance on wild Skeena sockeye CU productivity, if an effect indeed existed, we performed a retrospective power analysis [37]. Power is a function of the effect size, variance in the response, and sample size. Because the true effect size in nature was unknown, we calculated power over a range of plausible smolt effect sizes from 0 to −0.4, which correspond to a reduction in survival of 0 to 33% when enhanced smolt abundance is increased from 66 to 100 million smolts (as previously reported [15]). Each simulation we describe below was based on the actual sample size (327 stock-recruit pairs) and base parameter estimates from equation 2.

We simulated the number of adult recruits in each CU and year as:
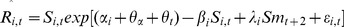
(3)where stochasticity, including random effects and environmental/measurement error, was incorporated into our power analysis using a bootstrap algorithm [38].

We then fit linear models with and without an enhanced smolt covariate (*λ*) to the simulated data and tested whether the smolt covariate model had greater statistical support than the null models based on a likelihood ratio test at *α* = 0.05. These simulations were repeated 10,000 times for each increment of the smolt effect size (0.01), and power was calculated as the proportion of the simulations where there was statistical support for the model with enhanced smolt abundance.

## Results

**Figure 4:**
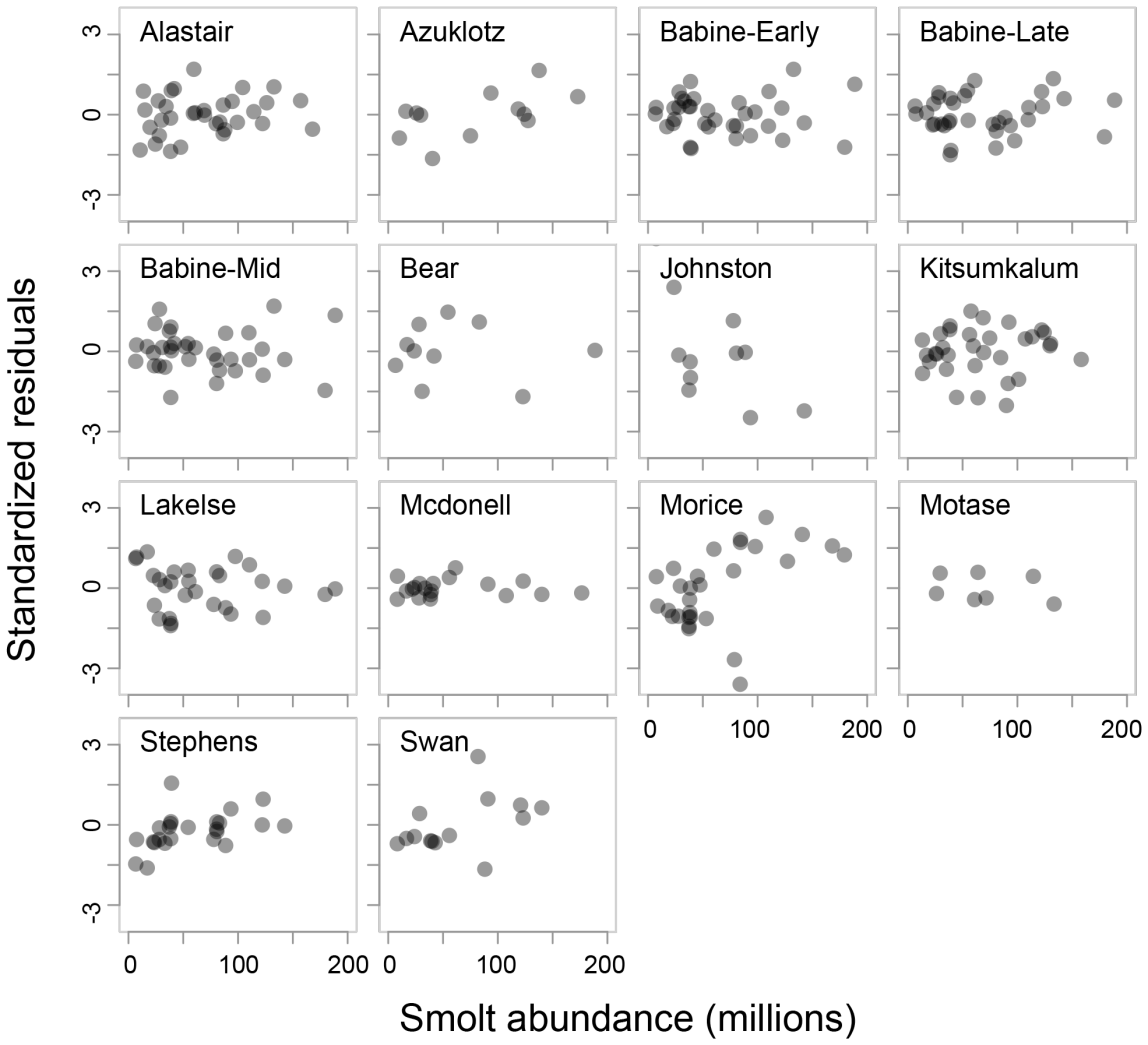
Productivity relative to smolt abundance for each wild Skeena lake-sockeye Conservation Unit. Legend: Standardized annual residuals are derived from the linear relationship between productivity [log_e_(*R_i,t_/S_i,t_*)] and spawner abundance, in relation to an index of the abundance of enhanced (late-migrant) sockeye smolts leaving Babine Lake.

**Figure 5:**
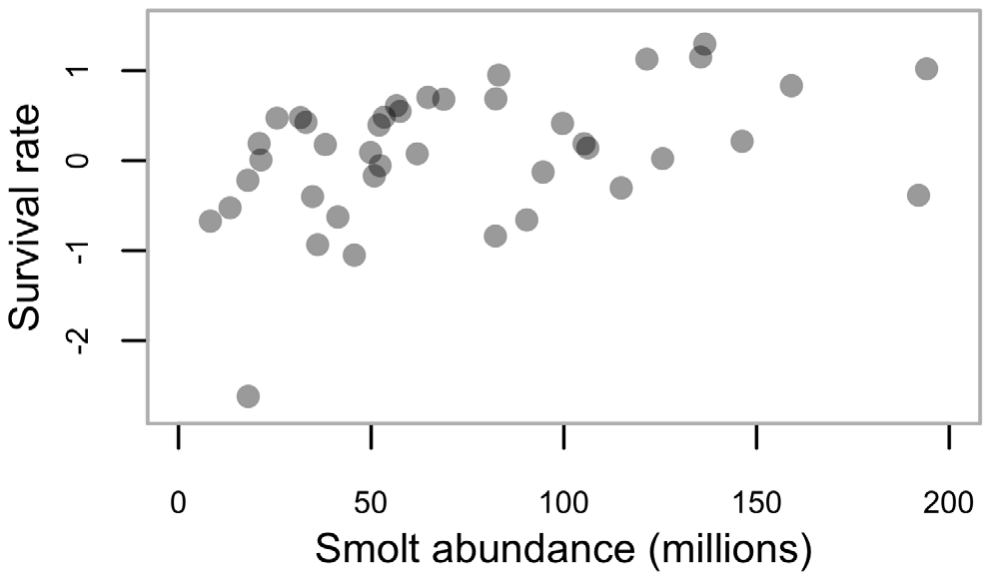
Productivity relative to smolt abundance for aggregate Babine (i.e., wild and enhanced) sockeye. Legend: Standardized annual residuals are derived from the linear relationship between productivity [log_e_(*R_i,t_/S_i,t_*)] and spawner abundance, in relation to an index of the abundance of enhanced (late-migrant) sockeye smolts leaving Babine Lake.

There was no obvious visual evidence of a relationship between Skeena (Figure 4), or aggregate Babine (i.e., wild and enhanced; Figure 5), sockeye productivity and the abundance of enhanced Babine Lake sockeye smolts in the year wild sockeye migrate to sea. We found little statistical support for the hypothesis that the productivity of wild Skeena sockeye salmon CUs is inversely related to the abundance of enhanced sockeye smolts leaving Babine Lake in the same ocean-entry year (Table 3). Instead, the null model of Skeena sockeye stock-recruit dynamics had the greatest statistical support, with some limited support for the hypothesis that the productivity of Skeena sockeye CUs productivity is negatively related to SST in the months preceding sockeye marine entry (Table 4). The null hypothesis was considered 1.7 and 9.8 times more likely than the hypotheses that included SST and enhanced smolt abundance, respectively (Table 3). These three top hypotheses had a combined weight of 0.97 indicating that, given the data, there is a 97% chance that at least one of the three models is the best explanation of the data among the hypotheses considered. Models representing the other hypotheses had very little support as illustrated by their large evidence ratios.

**Table 4:** Multi-model averaged parameter estimates and unconditional standard errors (SE) of parameters in the set of hypotheses considered.

Parameters	Coefficient	SE
*α*	1.771	0.099
SST	−0.159	0.063
Sm(f)	−0.004	0.122
Sm(f) x SST	−0.067	0.059
Sm(b)	−0.002	0.002
Sm(b) x SST	−0.001	0.001

Legend: Productivity (log_e_[recruits/spawner]) at low spawner abundance is *α*, and the variables are: enhanced Babine smolt abundance at the full Skeena (Sm(f)) and Babine Lake (Sm(b)) scale, sea surface temperature (SST), and an interaction between the two (x). All parameters were estimated from a dataset in standard deviation units (SDU) to permit meaningful comparisons because the independent variables are on different numerical scales. For example, the −0.159 parameter estimate for SST means that a 1 SDU increase in SST results in an decrease of 0.159 log_e_[recruits/spawner] or 0.85 recruits/spawner.

When model uncertainty was accounted for by calculating parameter estimates averaged over the support for the models in which they occurred, SST had the strongest predicted influence on Skeena sockeye productivity of the factors considered. A one standard-deviation-unit increase in SST is predicted to result in a decrease of 0.16 log_e_[recruits/spawner] units or 0.85 recruits/spawner (Table 4). The remaining parameter estimates were both very small in magnitude and highly uncertain.

**Figure 6:**
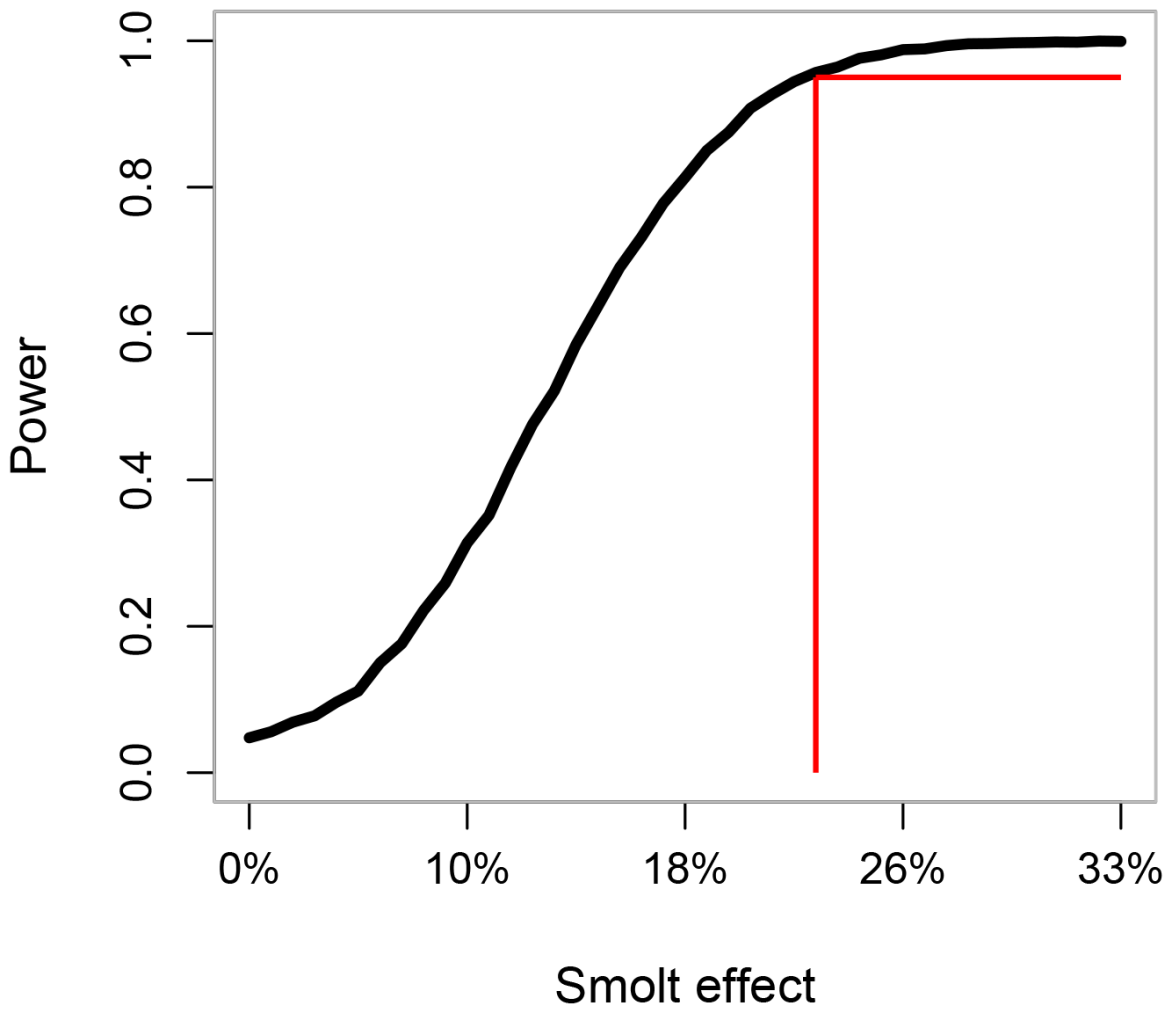
Statistical power to detect an effect of channel-enhanced sockeye smolt abundance. Legend: Power is the probability of correctly detecting some specified effect size at α = 0.05) to detect an effect of Babine smolt abundance on wild Skeena lake sockeye Conservation Units (CU). The “smolt effect” is the reduction in wild Skeena lake sockeye CU productivity due to an increase in smolt abundance from 66 to 100 million out-migrating smolts, and the red line is the “smolt effect” estimated from the relationship between Babine smolt-to-adult survival and out-migrating smolt abundance from 1962–2002 (Figure 2).

Previous analyses have illustrated an inverse relationship between smolt-to-adult survival and the abundance of Babine smolts [15], [20], [21] (our Figure 2), which we estimate corresponds to ∼22% reduction in smolt-to-adult survival when enhanced smolt abundance is increased from 66 to 100 million smolts (based on back-transforming the slope of the relationship between log_e_(smolt-to-adult survival), and scaled smolt abundance) [15]. Given the variability inherent in the data, and the number of stock-recruit pairs we had from the CUs examined, we had high statistical power (i.e., >80%) to detect an effect of Babine Lake smolt abundance as great or greater than what was previously estimated for the Babine system [15] (red line in our Figure 6), but weak power to detect smaller effects of Babine smolt abundance on wild Skeena sockeye productivity (i.e., <10% reduction in survival when enhanced smolt abundance increases from 66 to 100 million smolts).

## Discussion

It has long been known that smolt-to-adult survival for Babine Lake sockeye is inversely related to the abundance of sockeye smolts leaving Babine Lake [15], [17], [20], which has led to the concern that enhanced smolt production may also depress wild non-Babine Skeena sockeye populations through increased competition [21]. Despite this clear inverse relationship between aggregate Babine smolt-to-adult survival and smolt abundance, the results of our formal analyses do not support the hypothesis that wild Skeena sockeye productivity (which examines survival beyond the marine life-phase to include freshwater) is reduced in years when they migrate to sea with large numbers of enhanced Babine Lake smolts. The lack of support for this hypothesis could arise because there is not a negative effect of enhanced smolt abundance on wild sockeye survival (a true negative), or because the analyses failed to detect a negative relationship even though there is one (a false negative). The results of our power analysis suggest that we had high power to detect effects of enhanced Babine smolts on wild Skeena sockeye as great or greater than those previously described for Babine sockeye, but we had weak power to detect much smaller effects.

Other factors may exert a larger influence on the productivity of Skeena sockeye than Babine Lake smolt production. For example, there could be limited ecological overlap between enhanced and wild smolts either during freshwater emigration to the ocean, or during early marine rearing in the Skeena estuary. Any density-dependent effects that may be present are perhaps localized, are too small to be detected, or may not be measurable at the CU level. Given the paucity of information regarding spatial and temporal patterns of CU-specific migration and rearing, this is an important area of future research.

However, our failure to reject the null hypothesis that enhanced Babine smolts do not influence the survival of wild Skeena sockeye may be a false negative for at least two reasons. First, there is likely large variability in mortality processes captured in our estimates of productivity over the entire life span (i.e., recruits-per-spawner), such that any relationship between the productivity of wild Skeena sockeye and enhanced Babine Lake smolt abundance may be obscured. Ideally the analyses would have used an index for smolt-to-adult survival. But because smolt abundance for wild Skeena sockeye CUs and wild smolts from Babine Lake are not available, our analyses relied on an index of productivity that integrates over both freshwater and saltwater life stages. While most mortality for salmon is thought to occur during early marine life [39], [40], factors influencing life-history phases beyond the early marine stage, such as competition for open-ocean resources with other salmon species (as recently reported for Fraser River sockeye [41]) or during freshwater rearing with conspecifics (which may now be occurring within Babine lake [15]), may have a larger effect on Skeena sockeye productivity.

The strength of the correlation between recruits-per-spawner and smolt-to-adult survival for a given brood year is only moderate (for the Babine system), highlighting that estimates of survival based on adult recruits-per-spawner will only partially capture variation in smolt-to-adult survival. In addition, unlike for marine survival, there is not evidence of an inverse relationship between aggregate Babine (i.e., wild and enhanced) sockeye productivity and smolt abundance (Figure 5), which further suggests (at least in the case of Babine sockeye) that total life-cycle survival is likely an unreliable measure of the influence of smolt abundance on marine survival. Admittedly, marine survival only explains 24% of the variability in overall productivity, which implies that freshwater processes may be a significant factor in shaping the productivity of Skeena sockeye, at least in Babine Lake.

The second reason a false negative may occur is due to poor data quality. Errors in estimates of productivity could arise as a result of inaccurate or biased estimates of escapement, exploitation, or age-class structure; all of which make-up the productivity estimate in a given brood year. While our analyses assume that escapement is measured without error, this is not true. The average survey quality rating for the CUs we examined was, “Fair - an estimate of moderate reliability based on two or more visual inspections”, and the escapement estimates that comprise the dataset we used are based on the conversion of escapement estimates for indicator streams within a CU to total escapement by CU [24]. Estimated exploitation rates for each CU are also subject to numerous assumptions ranging from those necessary for the simple summation of annual catch estimates, to complex run-reconstruction that could (but not necessarily do) bias exploitation in time or space, in difficult to predict directions [24], which could then bias estimates of recruits either low or high in a given year. Furthermore, the majority of CUs considered in our analyses had spawners in a given year assigned to brood years based on estimates from only one or two measures of annual age data, and 5 of 14 CUs had no age data at all. By assuming average age proportions across years (instead of year-specific proportions), the resulting time-series of recruits are likely to be dampened (i.e., less variable than they should be) because this assumption may lead to incorrectly assigning recruits from large brood years to neighboring years while biasing high the number of recruits in small brood years [42]. Such dampening of the recruitment time-series, and hence productivity, may mask high inter-annual variability in survival attributable to competition with enhanced smolts.

Our analyses found weak support for a negative influence of warming ocean temperature early in marine life on Skeena sockeye productivity (standardized parameter estimate  = −0.16), which is opposite to the estimated influence of SST on Skeena sockeye productivity from previous investigations (i.e., +0.18) [25], [27]. This may arise because we relied on CU-specific data, while previous analyses have used aggregate Babine sockeye stock-recruitment data, or because we relied on a later span of years, and warrants further investigation.

If quantifying the influence of interactions between enhanced and wild sockeye is an important component of Skeena sockeye conservation and management, research programs designed to collect the necessary data to examine interactions between wild and enhanced sockeye in the Skeena are justified given the data limitations outlined above, including few estimates of smolt-to-adult survival and age-structure for wild sockeye salmon CUs. Important data gaps and directions for future research include: i) enumerate smolts and returning adults at a counting fence at several key wild Skeena sockeye indicator systems (the counting fence at Babine lake was re-initiated in 2013) to allow for estimates of fry-to-smolt and smolt-to-adult survival, ii) improve smolt and returning adult age-composition data for all Skeena sockeye CUs to better estimate returns for each brood year, iii) improve catch monitoring to increase accuracy of returning adult estimates, iv) mark channel-enhanced fry and determine degree of spatial, temporal, and ecological overlap with wild sockeye smolts throughout freshwater and early marine residency, v) examine the population dynamics of key prey species for sockeye in rearing lakes and the Skeena estuary, and vi) investigate the bioenergetics and rearing capacity of the Skeena estuary. We believe the most powerful test of the influence of enhanced smolt abundance on the productivity of wild Skeena sockeye salmon CUs would be to experimentally manipulate the number of enhanced sockeye smolts (e.g., [43]), followed by a comprehensive research program to quantify density-dependent wild smolt responses to enhanced smolts under variable conditions in the Skeena estuary.

While evidence has been mounting for over 20 years to suggest that fish hatcheries may have unintended genetic and ecological consequences for wild salmon [7]–[10], detecting clear effects on wild salmon due to spawning channel enhancement remains challenging in the face of a variable environment and incomplete datasets. The inverse relationship between sockeye smolt abundance in Babine Lake and subsequent marine survival of Babine sockeye during 1962–2002 suggests that enhancement from spawning channels may negatively influence sockeye abundance within Babine Lake through increased competition [15], [21]. However, when we examined whether this may also be the trend for other sockeye populations within the Skeena watershed, we did not find evidence for reduced survival in years of large enhanced smolt abundance; though total life-cycle survival appears to be an unreliable measure of the influence of smolt abundance on marine survival. Within the Babine system, marine survival only explains 24% of the variability in life-time productivity. This suggests that freshwater processes may be a more significant factor in shaping the productivity of Skeena sockeye than marine survival that may in part be influenced by channel-enhancement, if at all. Given the significant data gaps however, we caution that further research is needed before more definitive conclusions can be drawn.
